# Rapid Spectral Dynamics in Hippocampal Oscillons

**DOI:** 10.3389/fncom.2022.880742

**Published:** 2022-06-10

**Authors:** M. S. Zobaer, Carli M. Domenico, Luca Perotti, Daoyun Ji, Yuri Dabaghian

**Affiliations:** ^1^Department of Neurology, McGovern Medical Center at Houston, The University of Texas, Houston, TX, United States; ^2^Department of Neuroscience, Baylor College of Medicine, Houston, TX, United States; ^3^Department of Physics, Texas Southern University, Houston, TX, United States

**Keywords:** brain rhythms, oscillons, hippocampus, theta, spectral wave

## Abstract

Neurons in the brain are submerged into oscillating extracellular potential produced by synchronized synaptic currents. The dynamics of these oscillations is one of the principal characteristics of neurophysiological activity, broadly studied in basic neuroscience and used in applications. However, our interpretation of the brain waves' structure and hence our understanding of their functions depend on the mathematical and computational approaches used for data analysis. The oscillatory nature of the wave dynamics favors Fourier methods, which have dominated the field for several decades and currently constitute the only systematic approach to brain rhythms. In the following study, we outline an alternative framework for analyzing waves of local field potentials (LFPs) and discuss a set of new structures that it uncovers: a discrete set of frequency-modulated oscillatory processes—the brain wave oscillons and their transient spectral dynamics.

## 1. Introduction

### 1.1. Motivation

Brain waves are manifestations of synchronized neuronal currents widely used for describing neurophysiological activity (Fries, 2005; Buzsáki, 2011; Thut et al., 2012; Cannon et al., 2014). However, our understanding of these phenomena depends fundamentally on mathematical and computational tools used for analyzing the recorded Local Field Potentials (LFPs). Most computational methods are based on breaking the signal into a combination of basic components suggested by the study specifics, e.g., wavelet analysis is most appropriate for studying time-localized events, such as ripples or spindles (Battaglia et al., 2004; Bosnyakova et al., 2006; Sitnikova et al., 2009; Luijtelaar et al., 2011), whereas Fourier decomposition is used for describing the oscillatory patterns of LFPs (Roopun et al., 2008; Aru et al., 2015; Lozano-Soldevilla et al., 2016; Cole and Voytek, 2017). Since most techniques are backed up by a completeness theorem, it may appear that selecting a specific decomposition is only a matter of convenience. This, however, is not the case: given that physiological mechanisms of the LFP oscillations and their functions are not yet fully understood, the task of establishing a physically adequate description of the signal's structure is not idle (Kopell et al., 2010; Buzsáki at al., 2012; Sreenivasan and D'Esposito, 2019). One may draw here a historical parallel with the use of the Ptolemaic system, in which every movement of a celestial object could be decomposed into a sufficient system of epicycles (Hanson, 1960; Van der Waerden, 1974, 1982; Babb, 1977). However, it was the discovery of the heliocentric system that eventually revealed the physical laws governing planetary motion (Gallavotti, 2001).

### 1.2. Approach

Discrete Fourier Transform (DFT) converts data series into a superposition of discrete harmonics with fixed frequencies, proportional to a certain base frequency ω_0_ (Brigham, 1988). This built-in rigidity of the Fourier spectra leads to a well-known conflict between the temporal and the frequency resolutions, manifested in many fields, from biology to Quantum Mechanics, which limits the method's resolution (Folland and Sitaram, 1997; Grünbaum, 2003). In the following, we use an alternative technique—*Discrete Padé Transform* (DPT)^1^, which also converts data points into a superposition of harmonics. However, the DPT harmonics are free to change frequencies independently, adapting their values on a moment to moment basis through the Padé Approximation Theory algorithms (Baker and Graves-Morris, 1996; Bessis, 1996; Bessis and Perotti, 2009; Perotti et al., 2013, 2019; DeVito and Dabaghian, 2014; Perotti and Wojtylak, 2018).

**Figure 1:**
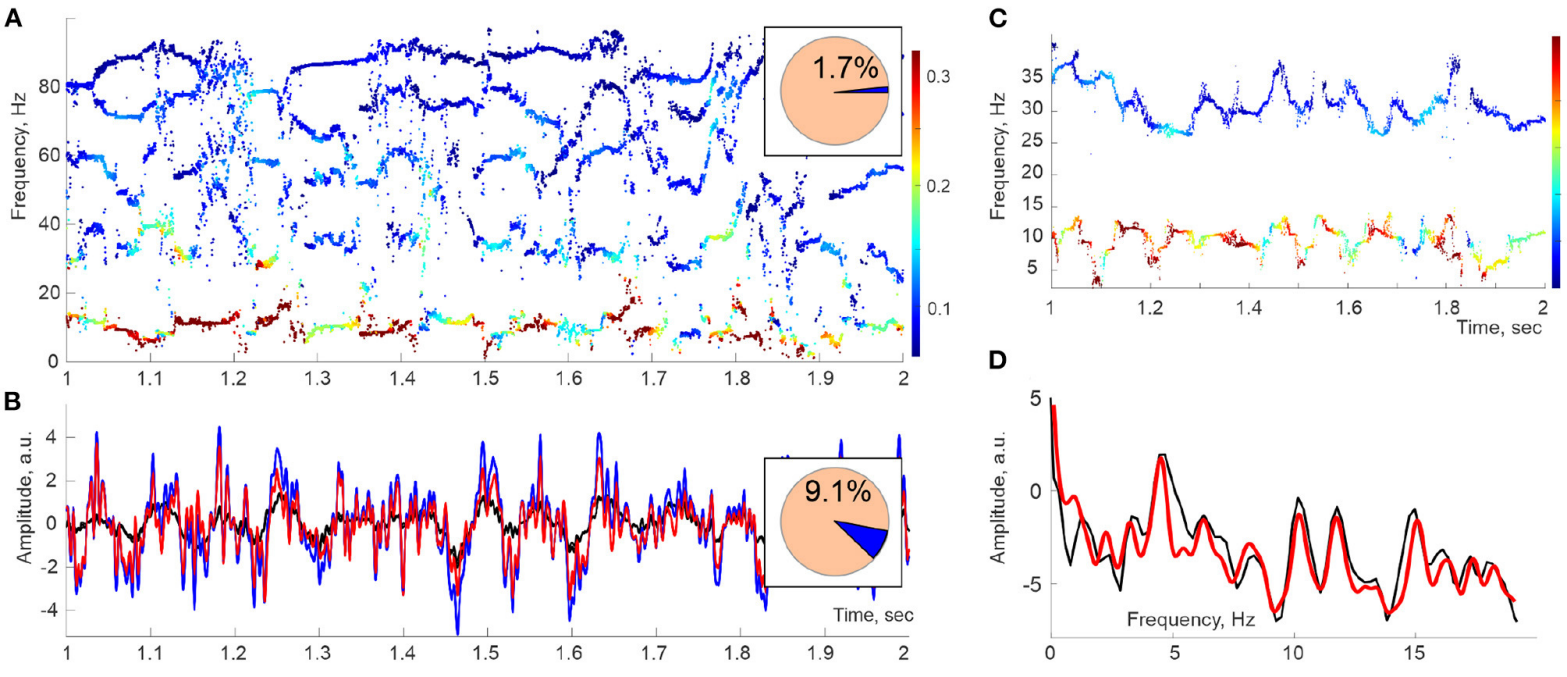
Spectral waves. **(A)** A second-long segment of the Discrete Pade' Transform (DPT) spectrogram computed for Local Field Potentials (LFPs) recorded in the hippocampal CA1 area of an actively moving rat exhibits a series of traces—the *spectral waves*, which can be viewed as timelines of time-dependent frequencies ω_*q*_(*t*). The dot colors designate the instantaneous amplitudes *A*_*q*_(*t*) of the corresponding oscillons (colorbar). Here, the sliding window width is *T*_*W*_ ≈ 50 ms, and the full number of DPT harmonics is *N* = 200, of which 1.7% are stable and produce spectral waves (pie diagram). **(B)** Oscillatory part of the LFP signal reconstructed from the stable frequencies (red) differs from the original signal (blue) by ≈ 9% of the signal's power (pie diagram). The mismatch is due to the discarded unstable frequencies, i.e., to the removed noise component ξ(*t*) (black curve). **(C)** At higher time resolutions (*T*_*W*_ ≈ 20 ms), spectral waves exhibit a quasiperiodic pattern. Shown are the θ (below) and the slow-γ (above) spectral waves. **(D)** The spectral power profiles constructed using Fourier (black line) and Welch's (red line) techniques show a set of peaks indicating the individual embedded frequencies.

The spectrograms of the LFPs recorded in the CA1 area of the rat's hippocampus, built using a “sliding window” version of DPT (refer to section 4), reveal patterns that open a novel perspective on the analyses of extracellular field dynamics. First, there appear to be two types of reconstructed frequencies. The first kind changes regularly across time, leaving distinct traces in the spectrogram—the *spectral waves* (Figure 1A). The frequencies of the second kind assume sporadic values from moment to moment and correspond to instantaneous “irregular” harmonics with much lower amplitudes. The nature of these two classes of harmonics can be explained based on several subtle theorems of Complex Analysis (Steinhaus, 1929; Froissart, 1973; Gilewicz and Pindor, 1997; Gilewicz and Kryakin, 2003). In essence, it turns out that the irregular harmonics represent the signal's noise component, ξ(*t*), whereas the regular, stable frequencies define its genuine oscillatory part, *r*(*t*) (Bessis, 1996; Bessis and Perotti, 2009; Perotti et al., 2013, 2019; DeVito and Dabaghian, 2014; Perotti and Wojtylak, 2018). Interestingly, the superposition of the regular harmonics, which typically constitute only 1 − 5% of the full set, captures the shape of the original signal with over 90 − 95% precision. Correspondingly, the contribution of the remaining 95 − 99% harmonics is small, typically less than 5 − 10% of the signal's amplitude (Figure 1B). Thus, according to the DPT, the brain waves consist of a few phase-modulated waves embedded into a weak noise background.


(1) $$\begin{array}{l} s\left(t\right)=\overset{M}{\underset{q=1}{\sum }}A_{q}e^{i\Phi_{q}\left(t\right)}+\xi \left(t\right). \end{array}$$

The individual oscillatory terms in (1), $\vartheta_{q}\left(t\right)=A_{q}e^{i\Phi_{q}\left(t\right)}$, are referred below as *brain wave oscillons* (DeVito and Dabaghian, 2014; Perotti et al., 2019).

Since the decomposition (1) emerges through empirical analyses, with no *a priori* assumptions or ansatzs, the oscillons may capture the physical organization of synchronized neuronal activity and help link empirical observations to theoretical models (Berger, 1933; Hoppensteadt and Izhikevich, 1997; Boashash, 2003; Vugt et al., 2007; Colgin, 2016).

Second, higher temporal resolutions reveal a quasiperiodic pattern of the reconstructed frequencies,


(2) $$\begin{array}{l} {\dot{\Phi }}_{q}=\omega_{q}\left(t\right)=\omega_{q,0}+\omega_{q,1}\sin(\Omega_{q,1}t\\ \;+\varphi_{q,1})+\omega_{q,2}\sin\left(\Omega_{q,2}t+\varphi_{q,2}\right)+\ldots , \end{array}$$

where ω_*q*,0_ is the mean frequency, and ω_*q,i*_ are the magnitudes of the embedded undulations with frequencies Ω_*q,i*_ and phases φ_*q,i*_ (Figure 1C). Importantly, the mean frequencies of the spectral waves dovetail with the mean frequencies of the traditional (i.e., Fourier-defined) rhythms. For example, the mean frequency of the lowest spectral wave (about 8 Hz) matches the mean θ-frequency and the mean frequency of the next spectral wave (about 32 Hz) aligns with the characteristic slow-γ frequency. Furthermore, the spectral undulation magnitudes are consistent with the widths of the corresponding Fourier bands (Senior et al., 2008; Carr et al., 2012; Colgin, 2015), which allows using the standard nomenclature, e.g., ω_θ_(*t*) for the spectral θ-wave, ω_γ_1__(*t*) for the spectral slow-γ wave, and to write the oscillon decomposition (1) in the form


(3) $$\begin{array}{l} s\left(t\right)=A_{\theta }e^{i\Phi_{\theta }\left(t\right)}+A_{\gamma_{1}}e^{i\Phi_{\gamma_{1}}\left(t\right)}+A_{\gamma_{2}}e^{i\Phi_{\gamma_{2}}\left(t\right)}+\ldots +\xi \left(t\right). \end{array}$$

The analysis of the spectral waves carried in Perotti et al. (2019) was motivated by the assumption that, in a given physiological state, the magnitudes ω_*q,i*_ and the embedded frequencies Ω_*q,i*_ in the expansion (2) are relatively stable and extractable through Fourier-based analyses, such as Welch's transform (Welch, 1967; Proakis and Manolakis, 1996). Indeed, the power profiles of approximately 1 s long segments of spectral waves exhibit consistent series of isolated peaks, suggesting that the hippocampal oscillons are driven by a discrete and comparatively scarce set of spectral harmonics (Figure 1D). However, further analyses revealed that the spectral dynamics are substantially more complex, as discussed below.

## 2. Results

**Figure 2:**
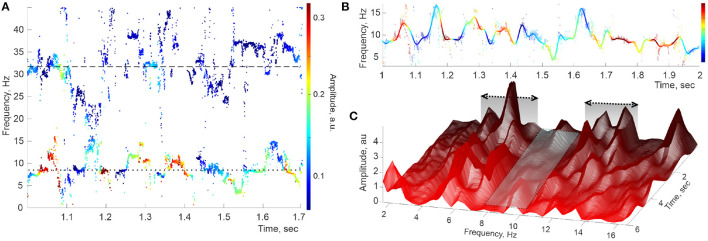
Hippocampal theta wave. **(A)** Stable frequencies falling under 40 Hz form intermittent traces occupying the θ-domain (lower trace) and the slow-γ domain (upper trace), with the means ω_θ,0_ ≈ 8 Hz and ω_γ_1_,0_ ≈ 32 Hz (doted and dashed lines, respectively). Color of the dots represents the instantaneous amplitude of the corresponding oscillons (colorbar). **(B)** Interpolating the raw θ-trace (dimmed pattern in the background) over uniformly spaced time points yields the reconstructed spectral wave ω_θ_(*t*) (solid colored line). **(C)** Welch's spectrogram of ω_θ_(*t*) exhibits domains of “peak ranges” (within the domains ΔΩ_θ_1__ ≈ 4 − 7 Hz and ΔΩ_θ_2__ ≈ 10 − 14 Hz), separated by a “valley” extending over ${\bar{\Delta \Omega }}_{\theta_{1}}=7-10$ Hz.

Since each instantaneous set of DPT frequencies is computed independently based on a finite number of data points, the resulting frequency patterns exhibit gaps and irregularities (Figure 2A). To capture the underlying continuous spectral dynamics (2), we reconstructed the contiguous pattern of frequencies and amplitudes by interpolating^2^ the “raw,” intermittent point traces over the full set of sampled times (Figure 2B). We then used the Welch's method (Welch, 1967; Proakis and Manolakis, 1996) to estimate the spectral density of the lowest spectral wave, ω_θ_(*t*). Specifically, about 12 s long LFP trace was split into Δ*t* ≈ 2.5 s long, highly overlapping segments, $\omega_{\theta }^{1},\omega_{\theta }^{2},\ldots ,\omega_{\theta }^{n}$, centered at times 𝔗 ≡ [*t*_1_, *t*_2_, …, *t*_*n*_],


$$\begin{array}{l} \omega_{\theta }^{i}=\omega_{\theta }\left(t\right),\text{for }t\in \left[t_{i}-\Delta t/2,t_{i}+\Delta t/2\right], \end{array}$$

with *t*_*i*+1_ − *t*_*i*_ ≈ 1 ms or less, and then Welch's procedure was applied to each segment. Arranging the resulting power profiles along the discrete time axis yields a three-dimensional (3D) spectrogram shown in Figure 2C, which demonstrates several curious features.

First, the lateral sections of the spectrogram—the instantaneous power profiles—exhibit a series of peaks, commonly situated within discrete frequency ranges (at ΔΩ_θ_1__ ≈ 4 − 7 Hz, then at ΔΩ_θ_2__ ≈ 10 − 14 Hz, then at ΔΩ_θ_3__ ≈ 16 − 19 Hz, etc.), separated by “valleys” in which peaks are rare (first extending over ${\bar{\Delta \Omega }}_{\theta_{1}}=7--10$ Hz, second over ${\bar{\Delta \Omega }}_{\theta_{2}}=14-16$ Hz, etc). This pattern was previously observed through static spectral power profiles such as the one shown in Figure 1D or in Perotti et al. (2019). The second surprising feature of the spectrogram is that most peaks are localized not only in frequency but also in time: a typical peak grows and abates over a few 100 ms periods. Third, many peaks are recurrent, appearing and disappearing repeatedly at about the same frequency Ω_θ,*i*_. Overall, the pattern illustrated in Figure 2C suggests that the oscillons' spectra are perturbed by a series of pulses that sporadically activate and wear off, as the animal navigates.

### Simulated Data

To validate the qualitative conclusion drawn from Figure 2C, we simulated a superposition of two oscillons with the spectral wave parameters derived from the recorded data. For example, the waves illustrated in Figures 2A,B were generated for the mean θ and γ frequencies, ω_θ,0_ ≈ 8 Hz and ω_γ,0_ ≈ 32 Hz, along with various specific sets of the reconstructed embedded frequencies, e.g., Ω_θ,*_ ≈ {1.9, 4.4, 6.2, 8.2, …} Hz and Ω_γ,*_ = {1.4, 3.9, 6.9, 9.2, …} Hz. These values were used to build “synthetic” θ and γ oscillons with spectral frequencies


(4θ) $$\begin{array}{l} \omega_{\theta }\left(t\right)=\omega_{\theta ,0}+\omega_{\theta ,1}\sin\left(\Omega_{\theta ,1}t\right)+\omega_{\theta ,2}\sin\left(\Omega_{\theta ,2}t\right)+\ldots , \end{array}$$

(4γ) $$\begin{array}{l} \omega_{\gamma }\left(t\right)=\omega_{\gamma ,0}+\omega_{\gamma ,1}\sin\left(\Omega_{\gamma ,1}t\right)+\omega_{\gamma ,2}\sin\left(\Omega_{\gamma ,2}t\right)+\ldots , \end{array}$$

which were then processed using DPT algorithms.

**Figure 3:**
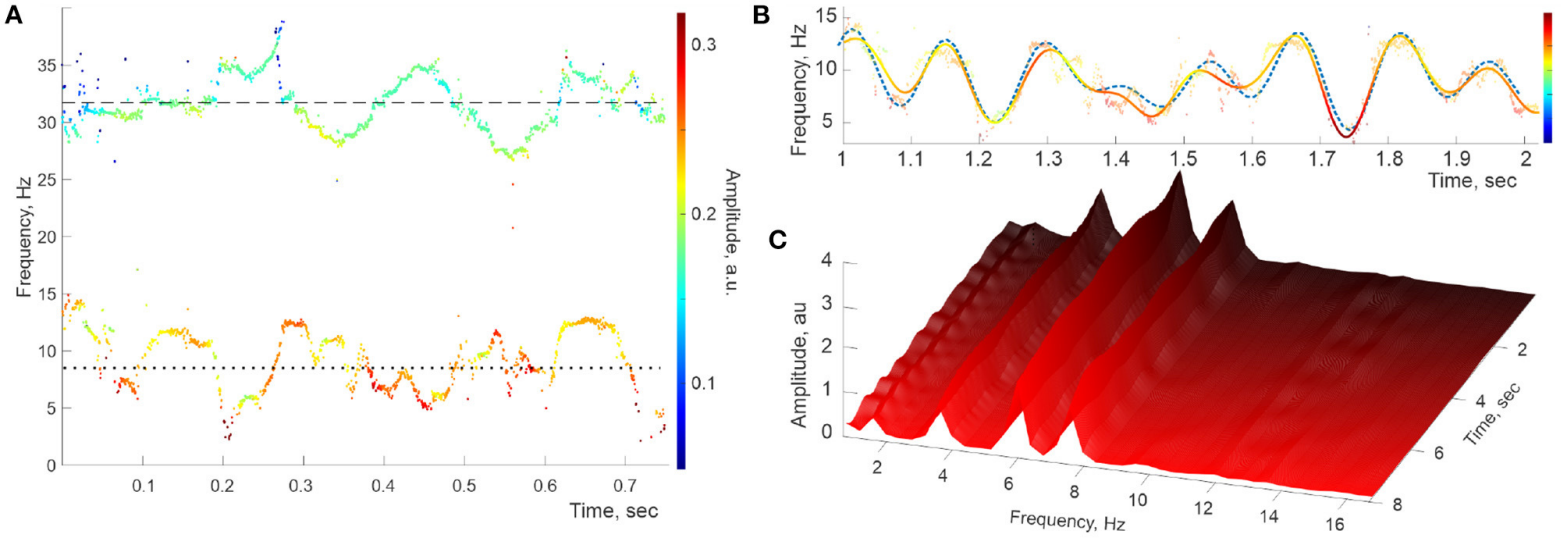
Simulated signal with constant embedded frequencies. **(A)** The power spectra of the simulated LFP wave—a combination of two synthetic oscillons produced at the same sampling rate as the recorded data. The timelines of the *reconstructed* stable frequencies undulate around the imputed mean values, ${\tilde{\omega }}_{\theta ,0}\approx 8$ Hz (dotted line) and ${\tilde{\omega }}_{\gamma_{1},0}\approx 32$ Hz (dashed line). **(B)** Interpolating the “raw” θ-trace (both frequencies and amplitudes) over the full set of timepoints yields the reconstructed spectral wave (solid colored line), which also matches the inputted ${\tilde{\omega }}_{\theta }$ (dashed line), with the embedded frequencies Ω_θ,1_ ≈ 1.9 Hz, Ω_θ,2_ ≈ 4.4 Hz, Ω_θ,3_ ≈ 6.2 Hz, and Ω_θ,3_ ≈ 8.2. **(C)** The “evolvent” Welch spectrogram reveals the embedded frequencies Ω_θ,1_, Ω_θ,2_ Hz, and Ω_θ,3_ that are sharply defined and approximately constant.

As expected, the stable frequencies reconstructed through DPT procedures produce clear point traces across the spectrogram, and the reconstructed contiguous spectral waves match the input data (Figures 3A,B). Correspondingly, the peaks representing the embedded frequencies appear in the Welch's spectrogram in correct positions and remain steady, nearly unchanged over the entire duration of the signal (Figure 3C). Furthermore, numerous computational experiments with synthetic oscillons produced no spurious peaks or other artifacts suggestive of the patterns visible in Figure 2C.

**Figure 4:**
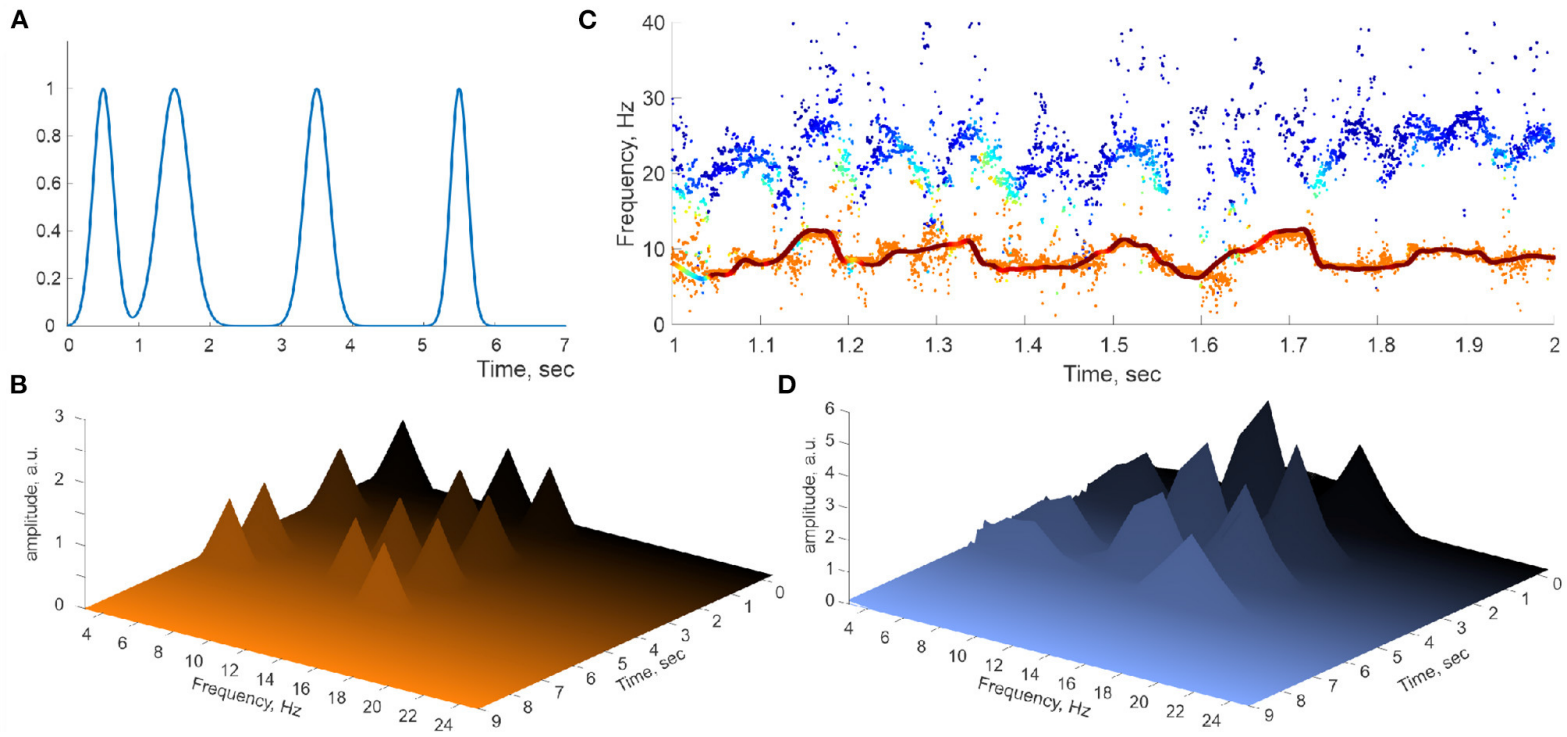
Perturbed spectral waves. **(A)** Series of Gaussian pulses applied to a simulated spectral harmonic. **(B)** Spectral pulses on Welch's spectrogram. **(C)** The corresponding spectral θ-wave (continuous line) and the underlying raw traces of the DPT-reconstructed stable θ and γ frequencies. **(D)** Welch's spectrogram of the spectral θ-waves reproduce the locations of the spectral pulses.

The ostensible difference between the spectrogram produced by the simulated oscillons with constant spectral waves (Figure 3C) and the ones reconstructed from the recorded LFP data (Figure 2C) suggests that the hippocampal extracellular field dynamics may not be described by quasiperiodic series (4a) with steady coefficients. The time-localized peaks visible in Figure 2C suggest that the hippocampal frequency spectra are disturbed by rapid, transient processes that appear for a short time and rapidly disappear. To verify this possibility, we applied the DPT analyses to a numerically generated signal in which the spectral waves with constant coefficients (4) were replaced by a superposition of harmonics with time-localized spectral magnitudes,


(5θ) $$\begin{array}{l} \omega_{\theta }\left(t\right)=\omega_{\theta ,0}+{\hat{\omega }}_{\theta ,1}\left(t\right)\sin\left(\Omega_{\theta ,1}t\right)+{\hat{\omega }}_{\theta ,2}\left(t\right)\sin\left(\Omega_{\theta ,2}t\right)+\ldots , \end{array}$$

(5γ) $$\begin{array}{l} \omega_{\gamma }\left(t\right)=\omega_{\gamma ,0}+{\hat{\omega }}_{\gamma ,1}\left(t\right)\sin\left(\Omega_{\gamma ,1}t\right)+{\hat{\omega }}_{\gamma ,2}\left(t\right)\sin\left(\Omega_{\gamma ,2}t\right)+\ldots , \end{array}$$

where ${\hat{\omega }}_{\theta ,i}\left(t\right)$ are narrow ($\sigma_{t}^{2}\approx 70-80$ ms) Gaussian pulses localized at a few discrete moments (Figure 4A). These “spectral kicks” are clearly manifested on Welch's spectrogram computed directly for the simulated spectral θ- and slow-γ waves (Figure 4B), but they do not significantly alter the reconstructed stable frequency traces (Figure 4C). Applying DPT analyses to the corresponding oscillons produces Welch's spectrograms that bear an uncanny resemblance to the spectrograms obtained for hippocampal LFP spectral dynamics (Figure 4D). The latter result suggests that the hippocampal oscillons may exhibit elaborate behaviors that include rapid, nonstationary spectral modulations that may be due to the extracellular field's endogenous dynamics or to inputs from parahippocampal or cortical networks.

## 3. Discussion

Discrete Fourier Transform techniques currently provide the most commonly used semantics and the main framework for interpreting the structure and physiological functions of the brain waves (Roopun et al., 2008; Buzsáki, 2011; Colgin, 2016). DPT offers an alternative, high-resolution technique that leads to a novel perspective on the LFP's oscillatory component, extracted from its “noise shell.” Specifically, DPT analyses indicate that the conventional, i.e., Fourier-defined θ, γ, and other brain waves conceal elaborate, frequency-modulated oscillatory processes—the oscillons, that may reflect physical dynamics of the extracellular fields.

The term “oscillons” is currently used in several fields, to designate, e.g., quasi-stable solutions of dynamic equations in field theory and cosmology (Gleiser, 1994; Copeland et al., 1995; Kasuya et al., 2003; Amin and Shirokoff, 2010) (also refer to Bogolubsky and Makhankov, 1976) or quasi-stationary undulations in granular media (Umbanhowar et al., 1996; Cerda et al., 1997). In this context, the physical origins of the brain wave oscillons require additional studies. Some properties of the oscillons' dynamics dovetail with predictions of theoretical models that aim to explain the coherent dynamics of extracellular fields through synchronization of neuronal activity in excitatory and inhibitory networks (Hoppensteadt and Izhikevich, 1998; Izhikevich, 1999a,b, 2000; Neda et al., 2000a,b). For example, the Kuramoto model of emergent synchronization (Strogatz, 2000; Arenas et al., 2008) describes networks of weakly interacting phasors with close natural frequencies ω_*i*_,


(6) $$\begin{array}{l} {\dot{\phi }}_{i}=\omega_{i}+\underset{j}{\sum }\lambda_{ij}\cos\left(\phi_{i}-\phi_{j}\right). \end{array}$$

As the coupling strengths λ_*ij*_ between the oscillators increase, the network transitions from a disordered to a partially synchronous and then to a globally synchronized state with a net phase


(7) $$\begin{array}{l} \Phi =\underset{i}{\sum }\phi_{i}. \end{array}$$

The form of the Equations (6) suggests that the expansion (2) should provide a natural ansatz for describing the functional form of the synchronized phase (7). Correspondingly, the initial analyses of oscillons (Perotti et al., 2019) were carried out under the assumption that spectral waves behave as almost-periodic functions with slowly varying coefficients, given that gradual changes of θ and γ bandwidths and their means, coupled to the animal's speed and acceleration, are well documented (Richard et al., 2013; Lu et al., 2020; Kropff et al., 2021). However, the current study suggests that oscillon dynamics involve not only slow but also rapid changes. In particular, it turns out that rapid dynamics affect not only the bandwidths and mean frequencies, but also the embedded frequencies, yielding time-localized “spectral pulses” that may reflect external inputs into the hippocampal CA1 area from other brain parts, e.g., from the hippocampal CA3 area or the medial entorhinal cortex (Brun et al., 2002; Kesner, 2007; Langston et al., 2010; Yamamoto and Tonegawa, 2017).

## 4. Methods

### 4.1. Discrete Fourier and Padé Transforms

Discrete Fourier Transform is produced by convolving the data values, *s*_1_, *s*_2_, …, *s*_*N*_, with a discrete set of harmonics with fixed frequencies,


(8) $$\begin{array}{l} A_{l}=\underset{n}{\sum }s_{n}e^{i\frac{2\pi l}{N}n}, \end{array}$$

arranged uniformly over the unit circle in the complex plane. The closer is the discrete frequency ω_*l*_ = 2π*l*/*N* to the frequency of the signal's constituent waves, $r\left(t\right)=\sum_{p}A_{p}e^{i\omega_{p}t}$, the bigger is the contribution of the corresponding harmonic into the decomposition (Brigham, 1988). If the data are sampled from a combination of harmonic oscillations and a noise background,


$$\begin{array}{l} s\left(t\right)=\underset{p}{\sum }A_{p}e^{i\omega_{p}t}+\xi \left(t\right) \end{array}$$

then each frequency ω_*p*_ produces a Fourier-peak, broadened and lowered by the noise ξ(*t*) (Newland, 2005; Perotti et al., 2014).

The DPT extends the expansion (8) from the unit circle into the complex plane, $e^{i\frac{2\pi l}{N}}\to z$,


(9) $$\begin{array}{l} S\left(z\right)=\underset{n}{\sum }s_{n}z^{n} \end{array}$$

where *z* is a generic complex number. For the oscillatory component of *s*(*t*), the sum (9), extended to infinity, yields a meromorphic function,


(10) $$\begin{array}{l} R\left(z\right)=\underset{n}{\sum }\underset{p}{\sum }A_{p}z^{n}e^{i\omega_{p}n\tau }=\underset{p}{\sum }\frac{a_{p}e^{i\varphi_{p}}}{1-z/z_{p}}, \end{array}$$

whose poles, $z_{p}=e^{-i\omega_{p}\tau }$, and residues, $a_{p}e^{i\varphi_{p}}$, define the frequencies, the amplitudes, and the phases of the contributing harmonics (Bessis, 1996; Bessis and Perotti, 2009; Perotti et al., 2013, 2019; DeVito and Dabaghian, 2014; Perotti and Wojtylak, 2018).

The sub-diagonal Padé approximant to (10),


(11) $$\begin{array}{l} R_{N}\left(z\right)=\frac{P_{N-1}\left(z\right)}{Q_{N}\left(z\right)}, \end{array}$$

rapidly approaches *R*(*z*) as the degree *N* of the polynomials *P*_*N*_(*z*) and *Q*_*N*+1_(*z*) grows, $R\left(z\right)=R_{N}\left(z\right)+O\left(z^{2N}\right)$ (Baker and Graves-Morris, 1996). In particular, the poles *z*_*p*_ of *R*(*z*) are approximated by the roots ζ_*q*_ of the denominator in (11), *Q*_*N*+1_(ζ_*q*_) = 0 (Bessis, 1996; Bessis and Perotti, 2009; Perotti et al., 2013).

As for the *z*-transform of the noise component,


$$\Xi \left(z\right)=\underset{n}{\sum }\xi_{n}z^{n},$$

the Steinhaus theorem establishes that its poles appear at the unit circle with unit probability (Steinhaus, 1929). The manifestation of this effect in the Padé approximations to Ξ(*z*) is subtle: the “noisy” poles are the ones that not only cluster around the unit circle, but also pair with the zeroes of Ξ_*N*_(*z*), thus forming the so-called “Froissart doublets” (Froissart, 1973; Gilewicz and Pindor, 1997; Gilewicz and Kryakin, 2003). A typical pole-zero distance in these pairs is smaller than 10^−6^ − 10^−7^ in the standard Euclidean metric in *C*^1^. Furthermore, the Froissart doublets are unstable with respect to variations of the algorithm's parameters, in contrast with the unpaired, stable poles produced by the regular part of the signal (Froissart, 1973; Bessis, 1996; Bessis and Perotti, 2009). These qualitative differences allow the separation of the regular component of the signal from its noise background, as expressed by the decomposition (1). The original study of Steinhaus (1929) presumed uniformly distributed noise series; subsequent works cited above allow generic, continuous noise distributions.

### 4.2. Sliding Window

Sliding window or the Short Time Padé Transform (STPT) uses a segment of the signal of length *T*_*W*_, centered at time *t*_*i*_, to extract the time-localized spectra—in full analogy with the Short Time Fourier Transform, STFT (Howell, 2001; Jacobsen and Lyons, 2003). Plotting the reconstructed frequencies along the vertical axis and arranging the times *t*_*i*_ horizontally yields the Padé spectrogram, which we use to illustrate spectral dynamics, in direct analogy with the standard Fourier spectrograms.

### 4.3. Signal Processing

The mean amplitude of the input data was normalized to $\overset{-}{s}\left(t\right)=2$. The LFPs were originally recorded at the rate *S*_*r*_ = 8 kHz. To increase time resolution in the biologically relevant range of frequencies (*f* <300 Hz), we interpolated the signal to higher rates (${\tilde{S}}_{r}=30$ kHz, ${\tilde{S}}_{r}=36$ kHz or ${\tilde{S}}_{r}=44$ kHz), which did not alter the shape of the studied spectral patterns but significantly improved stability and sharpness of the results. The oversampled time series were then downsampled 2 ≤ *m* ≤ 4 times, which produced *m* interlaced subseries that were independently studied with DPT. As one would anticipate, the stable frequencies generated by each subsequence form tight clusters of *m* points, grouping around the frequency produced by the original sequence, while the Froissart doublets exhibit erratic behavior (Bessis, 1996; Bessis and Perotti, 2009; Perotti et al., 2013, 2019; DeVito and Dabaghian, 2014). These procedures allow using time windows as short as *T*_*W*_ = 10 − −20 ms while keeping the order of the Padé approximants high, *N* = 100 or more. Shifting the time windows by a single data point ensures maximal contiguity of the reconstructed spectral waves and the oscillons' amplitudes. The Froissart distance used to identify close pole-zero pairs (Froissart doublet) is $d_{F}=10^{-6}$. To increase stability, the signals were filtered between 1 and 40 Hz

## Data Availability Statement

The data will be shared for research purposes once the corresponding papers have been accepted for publication.

## Author Contributions

YD conceived of the study, developed the method, carried analyses, and wrote the manuscript. MZ developed the method, conducted analyses, and provided figures. CD and DJ provided data and helped conceptualizing the results. LP developed the mathematical foundations of the method and helped adopting it to data analyses. All authors contributed to the article and approved the submitted version.

## Funding

MZ and YD are supported by NIH grants R01NS110806 and R01AG074226. CD and DJ are supported by NIH grants R01MH106552 and R01MH112523.

## Conflict of Interest

The authors declare that the research was conducted in the absence of any commercial or financial relationships that could be construed as a potential conflict of interest.

## Publisher's Note

All claims expressed in this article are solely those of the authors and do not necessarily represent those of their affiliated organizations, or those of the publisher, the editors and the reviewers. Any product that may be evaluated in this article, or claim that may be made by its manufacturer, is not guaranteed or endorsed by the publisher.

## Footnotes

^1^Throughout the text, terminological definitions are given in *italics*.

^2^The MathWorks, I. (2019). Symbolic Math Toolbox. Natick, MA, USA, see https://www.mathworks.com/help/curvefit/.
